# Juror characteristics on trial: Investigating how psychopathic traits, rape attitudes, victimization experiences, and juror demographics influence decision-making in an intimate partner rape trial

**DOI:** 10.3389/fpsyt.2022.1086026

**Published:** 2023-01-16

**Authors:** Caroline Lilley, Dominic Willmott, Dara Mojtahedi

**Affiliations:** ^1^School of Law, The University of Sheffield, Sheffield, United Kingdom; ^2^Division of Criminology, Sociology and Social Policy, School of Social Science and Humanities, Loughborough University, Loughborough, United Kingdom; ^3^Department of Psychology, School of Human and Health Sciences, University of Huddersfield, Huddersfield, United Kingdom

**Keywords:** jury decision-making, juror characteristics, rape myths, psychopathic personality traits, affective empathy, sexual victimization, juror ethnicity

## Abstract

**Introduction:**

Trial by jury is a longstanding legal tradition used in common law jurisdictions to try the most serious of criminal cases. Yet, despite hearing the same trial evidence, individual jurors often arrive at different verdict decisions, indicating that they may be impacted by more than the evidence presented at trial. This study therefore sought to investigate the role of jurors’ psychopathology, attitudinal, experiential, and demographic characteristics upon individual verdict decisions.

**Methods:**

Adopting an improved mock trial paradigm, 108 jury-eligible participants took part in one of nine identical 12-person mock trial simulations depicting a videotaped recreation of an intimate partner rape trial. Pre-trial, mock-jurors completed a psychosocial survey capturing their psychopathic personality traits (*affective and cognitive responsiveness, interpersonal manipulation; egocentricity*), rape myth beliefs, victimization experiences and demographics. Post-trial, jurors deliberated to reach a collective group decision and individual verdict decisions were recorded pre- and post-deliberation.

**Results:**

Binary logistic regression analyses revealed rape myth beliefs and juror ethnicity were significantly related to verdict decisions both pre- and post-deliberation. Post-deliberation, decreased affective responsiveness (empathy) and experience of sexual victimization were *also* found to be significant predictors of guilty verdict selections.

**Discussion:**

These findings indicate for the first time that within an intimate-partner rape trial, certain psychosocial traits, crime-specific attitudes, and experiences of sexual victimization appear to predispose juror judgments and decision-making even after group-deliberation. This study therefore has important implications for understanding how individual differences among jurors may impact rape trial verdict outcomes and the need for targeted juror reforms.

## 1. Introduction

### 1.1. Rape prevalence and attrition

Sexual violence against women is an epidemic-level global health crisis. Comprehensive global statistics now estimate that one in three women will experience an act of sexual violence during their lifetime (1). In England and Wales (E&W) alone, an estimated 773,000 adults reported some form of sexual assault in the year ending March 2020 (2), with an estimated 128,000 of those amounting to rape (3). Whilst it is acknowledged that both men and women can be victims of sexual violence, recorded crime data currently indicates that women are sexually victimized at almost four times the rate of men (2). However, it is important to recognize that men are found to face a range of additional barriers to reporting sexual violence which may impact these figures [see (4) and (5) for a detailed discussion]. Figures in E&W indicate that 98% of those prosecuted for the most serious sexual offenses were male, with females accounting for 84% of those victimized (6). Almost one-third of recorded sexual assaults are reported against a current or former intimate partner with global prevalence data concluding that 27% of women aged 16–49, who have been in an intimate relationship, reported experiencing sexual violence by their partners (1). One in two rapes are carried out by a current or former intimate partner in E&W alone (7). Clearly intimate partner rape (IPR) is a crisis overwhelmingly encountered by women (8).

A record high of 70,330 rapes were recorded by police in E&W in the year ending March 2022 (7). However, government estimates conclude that less than 20% of rape victims will ever report their experience to the police, indicating true prevalence is likely to be much greater (3). For the small percentage of victims who do report their experience, the likelihood of attaining a prosecution or conviction is extremely low. Recent UK data indicates that less than 2% of rape allegations result in an offender being charged (3) and a 2.4% point reduction in conviction rates between 2020 and 2021 (from 70.7 to 68.3%) (9). Indeed, it is well-documented that sexual assault and rape cases are far less likely to progress from perpetration to conviction than other criminal offenses. Notably, those committed by a current or former intimate partner generally attract much poorer rates of prosecutorial success in the criminal justice system (CJS) (10–12).

### 1.2. Rape myths in the criminal justice system

Vast theorizing has sought to understand why sexual violence continues to be perpetrated so frequently in an effort to explain prevalence and attrition rates within the CJS. Empirical evidence lends substantial support to the premise that widespread gender inequality and male dominance serve to normalize social and cultural acceptance of sexual violence against women and the misconceptions which surround sexual offenses (13–17). The prominence of factually incorrect, universally applied assumptions, beliefs, and attitudes which surround the circumstances of rape and sexual violence act as facilitators of societal ignorance toward such crimes and serve to normalize and misinform the public and professionals about the realities of rape, typically conceptualized as rape myths (18–20). Whilst studies show certain social groups are more likely to endorse these falsehoods (e.g., men, sports groups, offending populations), examining group differences based on demographics among a jury-eligible population, has rarely been considered. This is despite recognition of the pervasiveness of rape myths throughout global societies, and role that they play in attrition rates at each stage of the CJS, which in part, serve to deny victims access to justice or the pursuit of such (21–23). Rape myths are frequently observed within legal settings; with a plethora of empirical evidence reporting that police, judges, lawyers, and prosecutors utilize such myths in an effort to make sense of evidence within rape trials (24–30). Court observation research suggests that defense lawyers habitually exploit rape myths to influence juror perceptions of rape complainants’ credibility by relating specific case information to general “real rape” stereotypes (29, 31).

### 1.3. Rape myths and juror decisions

Unsurprisingly, the effects of rape myth endorsement upon juror judgments, decision-making, and deliberative discussions have been well-documented. Specifically, jurors who exhibit greater acceptance of rape mythology are significantly more likely to return not-guilty verdicts than those who endorse such beliefs to a lesser extent (27, 32–35). Kahan’s (36) early exploration of why jurors often appear to blame and disbelieve rape trial complainants concluded that individual juror characteristics could explain verdict selections. Adopting an experimental design among a large sample of 1,500 individuals who assessed a disputed allegation of rape, Kahan concluded that a hierarchical worldview (as opposed to an egalitarian viewpoint), predisposed assessors to agree with the defendants claim of belief in consent, despite the complainants repeated verbal objections. Indeed, numerous experimental mock-trial studies have sought to investigate the influence of rape myth beliefs upon varied aspects of juror decision making. Willmott et al. (30) asked mock jurors to complete pre-trial attitude questionnaires before exposing them to a video-taped acquaintance rape trial simulation. Before and after deliberating as a group, jurors were asked a series of questions assessing their belief in both the complainant and defendants’ testimony. Importantly, the authors found heightened rape myth acceptance scores were directly related to increased juror belief in the defendant’s version of events, though not with the account given by the complainant, where decreased belief in testimony was observed. According with the results of several other recent studies (25, 26, 33, 37), the findings indicate rape myth beliefs may predispose jurors to doubt rape complainants’ testimony though believe the defendant’s story of a consensual sexual encounter. In fact, a recent review of empirical studies examining rape case blame attributions found that irrespective of variation in the quality of the study design, greater endorsement of rape myths was consistently associated with complainant blame attributions in 28 of 29 studies [see (38)]. In a review of 28 separate studies Leverick (38) also found 25 empirical studies displayed direct evidence of a significant effect of rape myth beliefs upon the verdict decisions that juror made. Therefore, irrespective of variation in the methodological rigor and ecological validity found between mock jury studies, rape myth beliefs remain predicative of juror decisions.

Several high-quality qualitative studies have found evidence of the prejudicial influence that rape myths have upon mock jury decisions through examination of group deliberations. In an Australian context, Taylor and Joudo (39) conducted a series rape trial re-enactments involving a total of 210 mock jurors. Utilizing a genuine case the scenario involved a female complainant’s allegation of rape against a male work colleague. After deliberations, researchers discussed with jurors what factors had been most prominent in reaching their verdict and found wide variation in juror assessments of the complainant’s veracity, directly associated with mock juror perceptions of how a “*real*” rape victim would have behaved after an attack. The most prominent research examining the prejudicial influence of rape mythology upon rape trial jury deliberations was undertaken by Vanessa Munro and colleagues (40–43) across four mock-trial studies, three of which were live simulations high in ecological validity. In total more than 1,000 individual mock juries comprised within 107 deliberating mock jury groups were scrutinized. Though presented with differing rape trial scenarios, all trials were 75 min in length and all group deliberations lasted 90 min. Significantly, qualitative analysis for all four studies produced clear evidence that rape myths featured frequently throughout deliberations and often underpinned decisions to acquit. Deliberative discussions remained heavily focused upon the extent to which complainants exhibited clear physical resistance and the regularity with which women are perceived to make false allegations of rape. Such research findings have thereby drawn into question the ability of jurors to fairly and impartially evaluate evidence presented during rape trials, particularly with respect to IPR cases (sometimes referred to as domestic rape) where problematic attitudes surrounding a man’s perceived right to have sex with his partner or wife have historically been widely endorsed.

### 1.4. Psychopathic personality traits and juror decisions

To the authors’ knowledge, no published research is yet to examine how psychopathic personality traits (PPT), such as interpersonal manipulation (IPM) and egocentricity (ECO), may impact rape trial decisions made by individual jurors despite some evidence of a link between such traits and rape myth endorsement and sexually coercive behaviors. A review of the literature shows that PPT, typically those that reflect callousness and a lack of empathy, are broadly related to rape myth beliefs (44, 45). PPT that reflect traditionally maladaptive features of psychopathy have been found to be highly predictive of rape supportive beliefs (45). Interestingly, features such as callousness and cold-heartedness, are stronger in individuals who are sexually aggressive (44). Given evidence of an association between psychopathic traits and rape myth beliefs which often serve to doubt or disbelieve women’s experience of sexual violence, it seems appropriate to investigate for the first time in empirical research whether a direct relationship exists between distinct PPT and juror decision-making within the context of an adult IPR trial, using variable-centered techniques [see (35) for an alternative person-centered approach]. Particularly relevant given that a number of studies have shown juror empathy for a complainant directly influenced pro-victim judgments of discrete pieces of evidence during decision-making (46, 47). Compared to general attitudes regarding the law, how an individual perceives themselves and their ability to manipulate others has a clear relevance within the context of a deliberating jury. The ability of an individual to empathize with others, particularly those victimized in the case, may serve an important function within the decision-making process of final verdict outcomes.

#### 1.4.1. Operationalizing psychopathy

Despite disagreement operationalizing psychopathy, it has long been of interest within the CJS, especially as a tool to measure and explain criminal behavior. Cleckley (48) designed the earliest conceptualization of psychopathy, characterizing a “typical psychopath” by 16 traits, including superficial charm, unresponsiveness to interpersonal relationships, impulsivity, and antisocial behavior. Whilst psychopathy researchers continue to agree that it is indeed a multifaceted personality construct (49), it is now widely conceptualized as emerging in and beyond forensic settings and among varied non-offending societal groups (14, 50–52). In fact, many researchers now conceptualize the criminal and antisocial behavior feature of psychopathy to be a behavioral outcome of psychopathy personality rather than a core trait component [see (53, 54)]. As such, most existing psychopathy measurement tools have limited applicability outside of forensic and clinical populations. In response to this, the PPT Scale (PPTS) was created as a “clean” measure designed to capture psychopathy across four core components [IPM, ECO, affective responsiveness (AR), and cognitive responsiveness (CR)], regardless of an individual’s criminal background (51, 55). Such a tool provides a useful means by which the link between psychopathic traits and juror decisions can be explored among non-forensic community populations.

### 1.5. Demographic characteristics and juror decisions

Despite attempts to reliably measure the extent to which juror demographic characteristics hold any substantial influence over verdict decisions made at trial, literature yields inconsistent and contradictory results. Early research examining the role of juror age in verdict selections concludes that older jurors appear more conviction prone than their younger counterparts (56). More recently, researchers have found evidence of a link between the prevalence of guilty verdict preferences and increased juror age (57), thought to be the consequence of older population’s tendency to exhibit more favorable perceptions of law enforcement (58). Yet, other studies have failed to evidence any association between age and verdict decisions, with leading jury researchers concluding weak and inconsistent evidence supports the role of broad ranging demographic characteristics in juror decision-making (59). When considering gender, previous research has identified female jurors as significantly more conviction prone than males, particularly when seated on crimes of a sexual nature (46, 60), perhaps explained by the gendered nature of sexual victimization. Indeed, some studies conclude gender to be a direct predictor of verdict outcomes, with female jurors found to be approximately seven times more likely to return a guilty verdict than males (61). However, the empirical literature also remains unclear here with more recent evidence finding little to no differences between verdict decisions emerging as a product of juror gender (62).

It is perhaps unsurprising that a direct relationship has been identified between juror ethnic background and verdict decisions rendered at trial given the role of racial disparity among legal actors and those accused and victimized who find themselves in court. The evidence surrounding jurors favors a same-race leniency effect with evidence of greater conviction rates among defendants of a different race to jurors making such decisions (63–65). Research suggests a *reverse halo effect* that likens all negatively viewed racial group members to possess negative traits (64). Empirical research tends to focus on the role of juror race when the race of the defendant is manipulated. Therefore, there exists a gap in jury literature examining the role of a juror’s ethnicity upon verdict decisions where the race of the complainant and defendant are withheld. Regarding the role of jurors’ educational attainment upon verdict selection, research is somewhat limited. Debate and empirical investigation have centered upon the role of student vs. community sampled mock jurors in examining how differences between the two groups may influence the generalizability of findings to real world jurors [see (66)]. Whilst Hosch et al. (67) posits that well-educated jurors are likely to make well formulated calculated decisions that comply with legal instructions and demonstrate higher self-regard in their efforts to pursue fair and impartial trial outcomes, there remains a need for empirical research which seeks to understand the importance of juror educational attainment on verdict sections.

### 1.6. Sexual victimization and juror decisions

Finally, to date few empirical studies have explored the link between juror experience of sexual violence and verdict decisions made at trial. Whilst experiences of sexual abuse seem likely to influence juror judgments and verdict selections whilst serving as a rape trial juror, most existing studies found little evidence to support such an association (35, 68–70). That said, in one recent study, Bottoms et al. (71) sought to investigate the importance of jurors’ own abuse experiences on subsequent verdict selections when serving as jurors in a child sexual abuse trial. Here the authors did find evidence that sexual victimization experiences were associated with guilty verdict selections. Clearly, with a lack of clarity of empirical data surrounding adult rape trials and mixed findings in existing literature, the need to further elucidate the relationship between juror experiences of sexual violence and their verdict selections remains.

### 1.7. Study aims and rationale

This study aims to build upon limitations within existing jury research by developing an improved mock trial design that more accurately reflects the procedural stages present within genuine criminal trials. Most mock trial research fails to adhere to any standardized expectations of ecological validity with previous studies, typically assessing juror decision-making in isolation, without any inclusion of group deliberation or involvement of criminal justice personnel. Accordingly, previous research tends to reflect mere pre-deliberation verdict preferences rather than assessing juror verdict decisions after exposure to group deliberation–as occurs in genuine jury trials. This study thereby permits for the direct testing of the consistency by which pre-trial attitudes, victimization experiences and psychosocial traits ultimately influence initial and final verdict decisions. By closely simulating authentic jury procedures which reflect criminal trials in an English legal context, whilst retaining a level of methodological control and manipulation required within experimental research, the importance of juror characteristics within genuine IPR trials can be more reliably inferred. The study also intends to investigate for the first time, the role of juror PPT upon rape trial verdict decisions and examine group differences in rape myths among a population jury-eligible participants.

## 2. Materials and methods

### 2.1. Sample and sampling procedure

A self-selecting opportunity sample of 108 participants were recruited from a university campus in the North of England. Potential participants were targeted through advertisement posters (placed throughout the university campus) that displayed information about the experimental procedure and an email address to contact for participation. Individuals who responded *via* email received a web link to an event management website (*Eventbrite*) which presented a more detailed description of the experiment, participants’ expected role, and a platform to sign up for one of the mock trial experiment dates scheduled over a 4-week period. Potential participants were informed that all mock trials were identical and were asked to enroll on one date only, based on their preferred availability. They were also encouraged to do so in isolation rather than with their friends or known peers. All participation requests were vetted to ensure jury-eligibility in line with the current criteria in E&W. As such, all participants were (1) aged between 18 and 75, declared that they had (2) no serious criminal offense history, were not suffering from any (3) severe mental health illnesses, and (4) eligible to vote in local and government elections in the UK (i.e., they had lived in the UK for at least 5 years since their 13th birthday). Self-selecting participants who did not meet these jury-eligibility criteria were excused from taking part in the study. In total, 134 individuals responded to recruitment posters emailing the research team to express an interest in taking part. Of these, 11 were excused as they did not meet jury-eligibility criteria and a further 10 did not register after being sent the Eventbrite participation link. Of the 113 remaining participants who registered to take part in one of ten scheduled 12-person mock trials, nine trials went ahead with a total of 108 participants. The 10th scheduled mock trial was canceled as only five participants registered to partake.

The final sample of 108 participants were aged between 18 and 61 (*M* = 23.90, SD = 13.83) and were predominantly female (59.3%). A large percentage of the sample reported their ethnicity as Caucasian (63.9%), while the remaining 36.1% identified as black, South East Asian or from another minority ethnic group. As students were the target sample, differences in degree level were noted as education level. Hence, 75% of the total sample reported being in the process of completing an undergraduate bachelor’s degree, while the remaining 25% were enrolled on post-graduate programmes. Participants were also asked to disclose whether they had experienced a serious sexual offense, such as rape, of which 9.3% reported prior victimization experience (see Table 1 for full demographic details). Participants did not receive any reward or compensation for taking part.

**TABLE 1:** Descriptive statistics for age, AMMSA, and Psychopathic Personality Traits Scale (PPTS) subscales (*N* = 108).

Scale	*M*	SD	Observed min	Observed max
Age	23.90	7.88	18.00	61.00
AMMSA	90.89	22.35	37.00	135.00
AR	10.85	3.70	5.00	20.00
CR	10.56	3.17	5.00	19.00
IPM	13.63	3.87	6.00	23.00
ECO	12.98	2.99	6.00	22.00

AMMSA, acceptance of modern myths about sexual aggression; AR, affective responsiveness; CR, cognitive responsiveness; IPM, interpersonal manipulation; ECO, egocentricity.

### 2.2. Measures and mock trial materials

#### 2.2.1. Acceptance of modern myths about sexual aggression

The acceptance of modern myths about sexual aggression (AMMSA) scale (72) is a self-report unidimensional 30-item measurement tool designed to be a subtle measure of modern rape myth beliefs and broader attitudes held about sexual aggression (e.g., “women often accuse their husbands of marital rape just to retaliate for a failed relationship”). Responses are measured using a seven-point Likert scale (1 = “completely disagree” to 7 = “completely agree”) and are summed to attain a total rape myth score (possible range = 30–210), with higher scores indicating greater acceptance or endorsement of modern rape myths. The scale has received cross-cultural validation, having been found to demonstrate a reliable factor structure [e.g., (73, 74)] and satisfactory levels of retest reliability (between 0.67 and 0.88). The scale also demonstrated strong internal consistency within the present dataset (α = 0.92).

#### 2.2.2. Psychopathic Personality Traits Scale

The PPTS (55) is a 20-item self-report measure of PPT, designed to be used within diverse populations for research purposes. The scale was developed in response to the lack of existing measures that examined PPT within both offending and non-offending groups. The 20-item inventory measures psychopathic traits through four constructs: AR; 5 items, α = 0.86, which reflects the characteristics of low affective empathy and emotional shallowness (e.g., “What other people feel doesn’t concern me”); CR; 5 items, α = 0.76, which reflects an individual’s ability to understand the emotional state of others and emotionally engage with them at a cognitive level (e.g., “I am good at predicting how someone will feel”); IPM; 5 items, α = 0.84, which measures manipulative characteristics such as superficial charm, deceitfulness and grandiosity (e.g., “I sometimes provoke people on purpose to see their reaction”); and EGO; 5 items, α = 0.69, which reflects an individual’s tendency to focus on themselves, their own attitudes, beliefs and interests (e.g., “I believe in the motto: I’ll scratch your back, if you scratch mine”). Responses were measured on a 5-point Likert scale (1 = “strongly disagree” to 5 = “strongly agree”). For AR and CR factors, increased scores indicate a lack of each type of empathy, whereas higher scores on EGO and IPM factors indicate increased trait tendencies.

#### 2.2.3. Demographic information and verdict decisions

A self-report demographic questionnaire was devised to collect information regarding participants’ age, gender, ethnicity, current level of obtained education, and sexual victimization experience. Age was recorded as interval data, whilst all other demographic variables were recorded as nominal data. Ethnicity responses were recoded as Caucasian and Black-Asian Minority Ethnic ethnicity (typically abbreviated to BAME in the UK), due to a low cell count for individual non-Caucasian responses to allow for some form of comparison. Please note the term BAME is historically used to denote non-Caucasian British residents and citizens whose ethnic group is described as being of Black Caribbean or African decent or South-East Asian decent (typically Indian or Pakistani heritage). Recent census data suggests that BAME British residents represent 19% of the population of E&W (75). Verdict decisions to the question *“How do you find the defendant, on the allegation that he raped the complainant?”* were also binary coded as guilty/not guilty, according with genuine verdict categories used at trial.

#### 2.2.4. Mock trial materials

Case information in the current experiment was presented as a video-taped mock trial reconstruction that was filmed within a genuine Courtroom in the North of England. The case depicted was a genuine IPR allegation that was previously heard before a British court. A trial transcript was used develop the mock trial scenario presented in the following condensed structure: First, jurors were presented with the undisputed facts in the case, outlining an incident that occurred between one female and one male who were previously in an intimate relationship and who had met in the female’s apartment after the breakdown of the relationship where it is alleged the male raped the female. The male instead argues that whilst sexual intercourse did happen, this was consensual. Next, a summary of the prosecution case (key arguments) were presented to jurors, which included an audio recording of the complainant’s testimony (recreated by an actor), followed by a summary of examination-in-chief (by the prosecution) and cross-examination of the witness (by the defense). The main argument being that the defendant had wrongly assumed from their previous consensual intimate relationship that he was entitled to have sexual intercourse with the complainant and effectively did not carry out any steps to ensure that his belief in consent was reasonable. Next, the defendant’s account, again presented as an audio recording by an actor, followed by key arguments advanced by the defense and a summary of examination-in-chief, cross-examination, and re-examination. The main argument advanced by the defense was that the defendant had taken steps to ensure consent was obtained and therefore his belief was reasonable. The defense argue that the allegation of rape emerged because after sexual intercourse the defendant informed the complainant that he did not wish to re-enter into a long-term intimate relationship with her. Next, mock jurors were presented with a summary of the forensic evidence which outlined that whilst some evidence of injuries were found to the complainant’s genitals, it could not be determined whether these injuries were the result of consensual or non-consensual sexual activity. Finally, mock jurors were presented with the judge’s summary of the case and final instructions that jurors must follow (depicted by a trained lawyer who acted the role of the judge). Judge’s instructions were crafted in consultation with genuine trial transcripts, judicial guidance documents used by English judges when developing jury directions in real trials and in consultation with a genuine English Crown court judge. An expert panel comprising of senior police officers, prosecution and defense lawyers, and the aforementioned trial judge were consulted throughout the mock trial study to ensure that the reconstructions were closely aligned with UK legal practice and law of evidence. The final condensed video tapped trial was 22 min long.

### 2.3. Study procedure

A cross-sectional and experimental design was adopted whereby participants were recruited to take part in one of nine identical mock trial reconstructions held within a realistic mock courtroom within the law school at the host institution. Upon arrival to the experiment, participants were welcomed and asked to sit in the waiting area outside the mock courtroom and await further instruction until all 12 participants were in attendance. Each mock juror was provided with a study booklet upon entering the mock courtroom which contained an information sheet, consent form, experiment instructions, a battery of questionnaires (demographic questionnaire, AMMSA, PPTS, pre-deliberation verdict decision form, and post-deliberation verdict form). All questionnaires were completed in paper format and writing utensils were provided for each mock juror should they wish to take notes during the trial and deliberation, as is typical within English jury trials.

After providing informed consent, participants were asked to complete the demographic questionnaire, AMMSA, and PPTS. This process took participants between 20 and 25 minutes. Responses were collected and participants were informed that they would be watching the mock trial reconstruction video based on evidence presented from within a real trial. After the trial had concluded, individual mock jurors were asked to indicate their pre-deliberation verdict decision (guilty or not guilty) without discussing the case with their fellow jury panel members. Next, participants were presented with deliberation instructions. The mock jury panels were asked to appoint a jury foreperson among themselves to mediate and relay the collective verdict once deliberations had concluded. Jurors were informed that whilst they should aim to reach a unanimous verdict, if after 30 min they could not, a ten-two majority decision would be accepted. If a majority decision could not be reached, the foreperson would have to declare a hung jury. Participants were then asked to leave the jury box area of the court and directed to a large table on the other side of the mock courtroom to deliberate as a group. At this stage, the experimenter left the room to allow mock jurors to discuss the case openly and honestly. The foreperson was instructed to call the experimenter back into the room once a collective verdict had been decided. After doing so and without conferring, jurors were then asked to indicate their post-deliberation verdict decision. Participants were given explicit instructions that their final verdict decision *did not* have to reflect that of the collective jury but rather what they felt was the appropriate decision after deliberating with others. Finally, the elected foreperson was asked to announce the jury’s collective verdict. Participants were then informed that the trial was over, thanked for their participation and debriefed. Each experiment lasted between 120 and 180 minutes from arrival to debriefing.

### 2.4. Analytical procedure

All statistical analyses were performed using SPSS^®^ 26.0 (IBM Corporation, Armonk, NY, USA) for Windows^®^/Apple Mac^®^. For all logistic regression models, preliminary analyses indicated that there were no multivariate outliers and multicollinearity was unlikely to be a problem.

## 3. Results

Descriptive statistics for all scale data and frequency distributions for nominal data are presented in Tables 1, 2, respectively. Data pertaining to individual verdict decision frequencies indicates that at pre-deliberation, there was a slight overall preference toward guilty verdicts (55.6%), however, post-deliberation the distribution of verdicts evened out (Guilty = 50%). Collective verdict decisions differed with only two out of the nine juries returning a collective guilty verdict, four juries returning a not-guilty verdict, with three juries reaching a hung verdict. The disparity between individual juror decisions and collective jury-group decisions is likely explained by the requirement for at least a majority (ten jurors to two) collective decision. If this was not agreed upon during deliberations, either a not guilty verdict decision (where jurors perceived the defendant to be not guilty *or* were not sure of guilt beyond reasonable doubt) or hung jury (where jurors were unable to reach any decision at a ratio of at least 10-2) were required to be returned.

**TABLE 2:** Frequency distribution of nominal data (*N* = 108).

	Sample	Guilty verdicts pre-deliberation	Guilty verdicts post-deliberation
**Gender**			
Male	44 (40.7%)	20 (45.5%)	21 (47.7%)
Female	64 (59.3%)	40 (62.5%)	33 (51.6%)
**Ethnicity**			
Caucasian	69 (63.9%)	31 (44.9%)	32 (46.4%)
BAME	39 (36.1%)	29 (74.4%)	22 (56.4%)
**Degree**			
Postgraduate	27 (25.0%)	16 (59.3%)	14 (51.9%)
Undergraduate	81 (75.0%)	44 (54.3%)	40 (49.4%)
**Victim**			
Yes	10 (9.3%)	8 (80.0%)	9 (90.0%)
No	98 (90.7%)	52 (52.1%)	45 (45.9%)
Total	108	60 (55.6%)	54 (50%)

BAME, Black-Asian Minority Ethnic ethnicity.

### 3.1. Effects of deliberation

The data indicated that 22.2% of the sample (*n* = 24) changed their verdict after deliberation, with 8.3% of participants (*n* = 9) moving from not-guilty to guilty and 13.9% (*n* = 15) moving from guilty to not-guilty. The impact of deliberation was assessed using a procedure frequently used in jury decision-making research [e.g., (76, 77)]. A pre-deliberation-verdict certainty variable was created by multiplying participants’ initial verdict decision (−1 = not guilty; 1 = guilty) with their respective confidence score (1–10). Scores ranged from −10 (highly certain in not-guilty verdict) to 10 (highly certain in guilty verdict). The same procedure was used to create the post-deliberation-verdict certainty variable. Shapiro–Wilk test indicated that the data for both time points deviated from normality [*W*_pre–deliberation_ (108) = 0.784, *p* < 0.001; *W*_post–deliberation_ (108) = 0.785, *p* < 0.001], therefore, a Wilcoxon signed-rank test was used to compare verdict-decision certainty scores before and after deliberation. A significant change in verdict-decision certainty was observed between pre- (median = 5.5; IQR = 14) and post- (median = 1; IQR = 16) jury deliberation, *Z* = −2.47, *p* = 0.014. The results suggest that whilst, on average, there was still a preference toward guilty verdicts after deliberation, participants were significantly less confident in this decision. The change in verdict-decision certainty was small (*r* = 0.237), in accordance with Cohen (78).

### 3.2. Comparing sexually aggressive attitudes (AMMSA)

A Shapiro–Wilk test showed that AMMSA scores were normally distributed, *W* (108) = 0.99, *p* = 0.27 and suitable for parametric testing, thus, independent samples *t*-tests were conducted to compare AMMSA scores between gender and ethnicity groups.

The results indicate that males (*M* = 96.20, SD = 19.65) and females (*M* = 87.23, SD = 23.48), AMMSA scores differed significantly, (*t* = 2.151, df = 101.87, *p* = 0.034, *d* = 0.42). In relation to ethnicity differences, although BAME participants reported higher acceptance of sexually aggressive myths (*M* = 94.69, SD = 23.3), compared to Caucasian participants (*M* = 88.74, SD = 21.67), these differences did not reach statistical significance, (*t* = −1.306, df = 74.307, *p* = 0.195, *d* = 0.27).

### 3.3. Predicting verdict selections

Two binary logistic regression models were tested to determine the predictive effects of age, gender, ethnicity, education, previous sexual victimization, PPTS (AF, CR, IPM, and Ego), and rape myth acceptance (AMMSA) on verdict decisions both pre- and post-deliberation.

The model for pre-deliberation verdicts was statistically significant, [χ^2^ (df = 10, *N* = 108) = 37.83, *p* < 0.001, Cox and Snell *R*^2^ = 0.295, Nagelkerke *R*^2^ = 0.396], indicating that the model could distinguish participants who returned guilty verdicts from those that returned not guilty verdicts. The model as a whole correctly identified 71% of pre-deliberation responses. As displayed in Table 3, only AMMSA (OR = 0.95, *p* < 0.001) and ethnicity (OR = 0.16, *p* < 0.01) made a statistically significant contribution to the model. This indicated that participants who displayed decreased rape myth acceptance scores, and those that identified as BAME, were more likely to return guilty verdicts pre-deliberation, with the latter having a greater predictive effect.

**TABLE 3:** Binary logistic regression model for verdict decisions pre-deliberation (***N*** = 108).

Variables	*B*	SE	OR (95% CI)
AMMSA	-0.050	0.014	0.951*** (0.93/0.98)
AR	0.040	0.089	1.040 (0.87/1.24)
CR	0.145	0.099	1.156 (0.95/1.40)
IPM	0.054	0.073	1.055 (0.91/1.22)
EGO	0.064	0.097	1.066 (0.88/1.29)
Age	0.003	0.036	1.003 (0.93/1.08)
Gender (male)	0.195	0.554	1.215 (0.41/3.60)
Education (post-grad)	0.208	0.622	1.232 (0.36/4.17)
Ethnicity (Caucasian)	-1.811	0.593	0.163** (0.05/0.52)
Sexual victimization	1.551	1.029	4.716 (0.63/35.46)

SE, standard error; OR, odds ratio; 95% CI, confidence interval.

**p < 0.01 and ***p < 0.001.

A second test of the complete model was undertaken for verdict decisions made post-deliberation. The model was statistically significant, [χ^2^ (df = 10, *N* = 108) = 42.29, *p* < 0.001, Cox and Snell *R*^2^ = 0.32, Nagelkerke *R*^2^ = 0.42], indicating that the model could distinguish between individuals who returned guilty verdicts and those who returned not-guilty verdicts. The model explained correctly 79.6% of responses.

As displayed in Table 4, four variables made a statistically significant contribution to the model with the strongest predictor being previous sexual victimization (OR = 20.42, *p* < 0.05), however, this is likely to have been inflated by the small case size (*n* = 10). The next strongest predictor of a guilty verdict was ethnicity (OR = 0.32, *p* < 0.05), followed by AR (OR = 1.23, *p* < 0.05), and AMMSA scores (OR = 0.95, *p* < 0.001). The findings suggest that participants who were previously a victim of sexual violence, identified as BAME, exhibited increased scores in AR and exhibited reduced rape myth acceptance scores, were those most likely to return guilty verdicts following jury deliberation.

**TABLE 4:** Binary logistic regression models for verdict decisions post-deliberation (***N*** = 108).

Variables	*B*	SE	OR (95% CI)
AMMSA	-0.051	0.014	0.950*** (0.92/0.98)
AR	0.203	0.095	1.225* (1.02/1.48)
CR	0.041	0.097	1.042 (0.86/1.26)
IPM	0.018	0.075	1.018 (0.88/1.18)
EGO	-0.101	0.097	0.904 (0.75/1.09)
Age	-0.014	0.038	0.986 (0.92/1.06)
Gender (male)	-0.392	0.580	0.676 (0.22/2.11)
Education (post-grad)	-0.096	0.620	0.908 (0.27/3.06)
Ethnicity (Caucasian)	-1.143	0.577	0.319* (0.10/0.99)
Sexual victimization	3.016	1.341	20.418* (1.48/282.62)

SE, standard error; OR, odds ratio; 95% CI, confidence interval.

*p < 0.05 and ***p < 0.001.

## 4. Discussion

The main aim of this experiment was to investigate the impact of individual jurors’ psychopathic personality traits, rape myth beliefs, sexual victimization experiences, and demographic characteristics, upon verdict decisions made within IPR trials, both before and after group-deliberation. A secondary aim was to examine whether group differences were observed in rape myth acceptance scores among this jury-eligible sample, based on key demographic variables hypothesized as important in the context jury decision-making. In order that findings emerging from this study may be considered reliable and provide insights within genuine jury trials, the mock trial paradigm adopted was designed to more closely replicate authentic trial procedures within an English legal context. Regarding the main study aim, the combination of juror characteristics tested in this study were found to distinguish between mock jurors who returned guilty vs. not-guilty verdicts, both pre-and post-deliberation. These findings thereby provide support for the proposition that individual verdict decisions may be influenced by pre-existing juror biases and characteristics. Furthermore, findings were able to differentiate between traits and characteristics that appear to be influenced by group deliberation; that is, affective responsiveness/empathy and previous sexual victimization–found to impact decision-making only after group deliberative discussions had taken place. Clearly the importance of jury deliberation in the formation of final verdict decision-making processes among jurors cannot be underestimated. Whilst results infer that some juror characteristics have a consistent pre-dispositional influence and appear robust to deliberative discussions, others appear more sensitive to group deliberations. Also, the current findings evidence a direct relationship between rape myth beliefs and juror decisions in that, jurors who exhibited reduced endorsement of such falsehoods, were more likely to return a guilty verdict than their juror counterparts who exhibited greater belief in rape mythology–despite observing identical evidence during the trial. This finding accords with a wealth of previous research that also displayed such problematic beliefs appear to impact the impartiality juror’s evaluation of evidence and, ultimately, their determinations of guilt (28, 33, 38). The consistency with which rape myths were found to influence juror verdict selections pre- and post-deliberation appears to indicate that this form of crime-specific attitude may be robust during group-deliberation, with pre-trial juror perspectives seemingly unaffected by alternative views expressed by other jury group members. This is, however, somewhat speculative and future research may seek to explore this assumption through qualitative analysis of deliberative discussions among the jury panels [For a recent scoping review of the six common rape myths which pertain to IPR see (79)].

Juror ethnicity was also found to be a significant predictor of juror verdict selections, pre-and post-deliberation whereby jurors who self-reported their ethnicity as White/Caucasian, were less likely to return a guilty verdict than mock jurors whose self-reported ethnicity was Black African-Caribbean, South East Asian or from another minority ethnic background. As the literature surrounding the impact of juror ethnicity on decisions made at trial is sparse, this is a novel finding that requires further exploration in future research. Most previous research that has sought to explore the effects of juror race in so far as it is similar or dissimilar to that of the defendant/complainant’s ethnicity. Here studies tend to observe an apparent same-race leniency bias for defendants accused of serious crimes, until evidence is perceived as so damning that jurors of the same race become more punitive than those of a dissimilar race seemingly to distance oneself race from the actions of an individual (63, 65, 80). Importantly, to control for such an effect, defendant, and complainant race were purposely withheld from the current mock jury sample. In doing so, Caucasian jurors were less likely to convict despite exhibiting slightly lower rape myth beliefs overall. However, given that Black African-Caribbean, South East Asian, and mock jurors from other minority ethnic groups were not proportionally represented in the current sample, this finding should be interpreted with caution with future research seeking to re-examine the role of ethnicity among more racially diverse jury samples.

Regarding the role of juror’s psychopathology upon rape trial outcomes, of the four core components of psychopathic personality [as conceptualized (49, 51, 54)], affective responsiveness (AR) was found to be associated with jurors’ post-deliberation verdict selections. Here, those who exhibited heightened scores in (lack of) AR (i.e., displayed reduced affective empathy), were more likely to return guilty verdicts within the context of an IPR trial. One possible interpretation of this finding may be that for such jurors, deficits in affective empathy were directed toward the defendant in so far as the consequences of a delivering a guilty verdict that results in a lengthy prison term, whether fairly given or not, simply did not concern them. Indeed, there is evidence to suggest that for many rape trial jurors, the decision to return a guilty verdict is a burdensome one and emotions surrounding the consequences of a rape conviction on a man’s future seem paramount (35). Research on use of the not proven verdict in the Scottish jury system appears to support this assertation, with jurors seemingly choosing to “opt out” of the need to make a difficult decision especially in rape trials, in part possibly due to empathy held for the defendants’ circumstances who may otherwise be convicted [for a review see (81, 82)]. It is important, however, to note that the role of psychopathic personality traits upon juror decision-making is a new and a novel exploration within the current study; resultantly, further research is needed among varied case types and rape trial scenarios before definitive conclusions can be drawn surrounding the impact of such traits. Indeed, it stands to reason that a lack of empathy, the tendency to manipulate others, and the tendency to prioritize ones own thoughts and opinions above those of others may in fact have more prominence in the context of deliberating jury-group interactions. Future research should seek to examine the manifestation of such traits during high-stake jury group verbal and social interactions.

Finally, results indicate that sexual victimization experiences may directly influence juror decisions in that, post-deliberation jurors who reported having personally experienced a serious sexual offense were more likely to reach a guilty verdict than their non-victimized counterparts. This is a potentially important finding given the prevalence of sexual violence throughout the western societies from which jurors are typically drawn. The findings also correspond with results obtained by Bottoms et al. (71) who found that mock jurors with personal experience of childhood trauma and abuse, were more conviction prone than jurors without such experience. Though not all research adheres to such a conclusion with several studies failing to evidence any such association between personal victimization and juror voting preferences (68–70, 83). An important consideration within the current study was the low frequency of participants who reported such previous sexual victimization experiences, and thus a larger cross-section of sexual abuse survivors may yield different findings. To further elucidate the importance of sexual victimization upon juror decisions, future research should make use of a broader range of rape trial scenarios to better understand the parameters of any such effect and should seek to draw such inferences where a larger cohort of jurors with such experiences are included in the sample.

### 4.1. Strengths and limitations

Ultimately, where legal restrictions prohibit experimental research from being conducted with genuine jurors, psycho-legal research is limited in its ability to accurately mirror genuine trial environments (i.e., courtrooms, jury rooms), procedures (jury deliberations), and participants (genuine jurors from real-world criminal trials). Because of this, some policymakers and legal practitioners argue that psycho-legal research, such as mock or simulated jury trial settings that make use of written trial vignettes or scenarios, should not be consulted when determining the need for trial reforms (84). To address such criticisms, this study sought to improve the ecological validity of the mock trial procedures employed by implementing some of the minimum requirement recommendations suggested by Willmott et al. (23). As such, this study sought to make use of genuine case information and employed the services of professional criminal justice practitioners and actors in recreating the trial jurors were asked to decide upon. Mock jurors were presented with complete coverage of a trial, albeit condensed for feasibility purposes. The videotaped mock trial included in-depth information surrounding both complainant and defendant testimony, cross-examination, and legal instructions that match those presented to actual trial jurors in E&W. Indeed, use of a video trial in place of more commonly used written trial vignettes was in itself an improvement upon many mock trial designs. The inclusion of legal professionals in the development of trial materials and scrutiny of the delivery of such to mock jurors, was a further effort to improve the ecological validity of the study design and therefore, increase reliability and applicability of the emergent findings. However, by far the key strength of the mock trial paradigm employed in this study was the inclusion of group deliberation, a component frequently missing from existing mock jury research. Existing research typically assess individual decisions made in isolation of any deliberative discussions and therefore lacks the reality of the context within which genuine trial jurors reach their decisions. Given the improved mock trial design, the findings obtained may therefore provide a more accurate and reliable insight into the range of factors that influence criminal trial decision-making. That said, this study is not without limitations. Firstly, our use of a student sample. Whilst some prior research has concluded that there are unlikely to be significant differences in the decision-making of students and community samples (85), the experiences, educational attainment and attitudes of students clearly differ from those found to be more variable among the general population. The racial diversity of the sample was also limited and not representative of the varied ethnic groups which exist throughout the UK. The need to replicate this study among a more representative and diverse jury participant pool is therefore warranted. The size of our sample is a further limiting factor. However, whilst the study would have benefited from a larger overall sample size to generate greater statistical power, given the small effect sizes that were observed for the null findings, it is unlikely that a larger sample will have changed the trajectory of our findings. Furthermore, inspection of the literature signifies that our sample was adequately sized relative to similar existing studies on juror decision-making [e.g., (86–88)], likely based on the difficulty researchers face when attempting to recreate an in-person mock jury trial study such as this. This is evident from the scarcity with which such studies appear in the literature in place of the more common mock-juror studies conducted cross-sectionally online and without any group deliberation.

### 4.2. Implications

Based on evidence that varied juror characteristics do appear to predispose verdict decisions both pre- and post-deliberation, legislative restrictions that prohibit the experimental study of possible prejudicial bias among genuine jurors, ought to be relaxed. Greater researcher access to the decision-making process of genuine trial jurors will allow for more reliable and conclusive testing of the extent to which such factors have any negative impact upon juror fairness and impartiality. Where access to genuine jurors is not permitted, future research would benefit from seeking to collectively adhere to improve mock trial paradigms, exhibiting heightened external, and ecological validity. In doing so, where minimum standard thresholds are adopted (see 23), study findings are more readily comparable and useful to policy makers seeking to evaluate the range of jury reforms being advanced.

## 5. Conclusion

The current study aimed to examine how a range of factors including, rape-myth beliefs, psychopathic personality traits, juror demographics, and sexual victimization experiences may influence and explain variability in IPR trial juror decisions. Findings clearly indicate the presence of a relationship between pre-trial characteristics and juror decisions–both pre- and post-deliberation. Practically, the implications of these findings are clear and multifaceted. Where rape myths and other pre-trial juror characteristics predict verdict selections even following group deliberation, the impartiality of juror decision-making is drawn into question. Taken together these findings raise serious questions about the fairness of juror and jury decisions within contested rape trials and rightly reignite debate surrounding the need for jury reforms to tackle such prejudicial influence on rape trial outcomes.

## Data availability statement

The raw data supporting the conclusions of this article will be made available by the authors, without undue reservation.

## Ethics statement

The studies involving human participants were reviewed and approved by the School Research Ethics Committee, University of Huddersfield. The patients/participants provided their written informed consent to participate in this study.

## Author contributions

All authors contributed equally to each stage of the study design, data collection, analysis, and write-up.
